# Role of imaging techniques in monitoring atrial cardiomyopathy and atrial failure: a scientific statement

**DOI:** 10.1093/eschf/xvag068

**Published:** 2026-03-03

**Authors:** Massimiliano Camilli, Emilia D’Elia, Jean Sebastien Hulot, Massimi Iacoviello, Nicolò Sisti, Otilia Tica, Mariya Tokmakova, Jef Van den Eynde, Jerremy Weerts, Rosita Zakeri, Antoni Bayes Genis, Wojciech Kosmala

**Affiliations:** Department of Cardiovascular Medicine, Fondazione Policlinico Universitario A. Gemelli IRCCS, L.go A. Gemelli, Rome, Italy; Department of Cardiovascular and Pulmonary Sciences, Catholic University of the Sacred Heart, Rome, Italy; Cardiovascular Department, Cardiology Unit, ASST Papa Giovanni XXIII, Bergamo, Italy; School of Medicine and Surgery, University Milano Bicocca, Milano, Italy; Clinical & Translational Research Center, PARCC INSERM U970 European Hospital Georges Pompidou APHP, Paris, France; Department of Medical and Surgical Sciences, University of Foggia, Foggia, Italy; Department of Cardiology, Hospital of Gubbio-Gualdo Tadino, Gubbio, Italy; Bihor County Emergency Clinical Hospital, Cardiology Department, Oradea, Romania; Medical University of Plovdiv, First Department of Internal Diseases, University Hospital for Active Treatment ‘Sv. Georgi’ EAD, Plovdiv, Bulgaria; University Hospitals Leuven, Department of Internal Medicine, Leuven, Belgium; Cardiovascular Research Institute Maastricht and Maastricht University Medical Center, Cardiology Department, Maastricht, The Netherlands; School of Cardiovascular Medicine & Metabolic Sciences, Faculty of Life Sciences and Medicine, King’s College London, London, UK; ICREC Research Program, Germans Trias i Pujol Research Institute (IGTP), Badalona, Barcelona, Spain; Heart Institute (iCOR), Germans Trias i Pujol University Hospital, Badalona, Barcelona, Spain; CIBER Cardiovascular, Instituto de Salud Carlos III, Madrid, Spain; Department of Medicine, Autonomous University of Barcelona, Barcelona, Spain; Institute of Heart Diseases, Wroclaw Medical University, Borowska 213, Wroclaw 50-556, Poland

**Keywords:** Multimodality imaging, Atrial cardiomyopathy, Atrial failure, Heart failure, Atrial fibrillation, Monitoring

## Abstract

Atrial cardiomyopathy (AtCM) is increasingly recognized as a distinct pathological entity characterized by structural, functional and electrical abnormalities that may predispose to atrial fibrillation, heart failure and other adverse cardiovascular outcomes. Early identification and longitudinal monitoring of atrial remodelling are therefore crucial to improve risk stratification, guide therapeutic decisions, and assess treatment response. However, clinical evaluation alone is often insufficient to capture the complexity and temporal evolution of atrial disease. Multimodality cardiac imaging plays a central role in the detection, characterization and surveillance of AtCM and atrial failure, the latter representing the advanced stage of this continuum. This scientific statement synthesizes the current evidence supporting the use of imaging techniques for monitoring AtCM across diverse clinical scenarios. The strengths and limitations of echocardiography, cardiac magnetic resonance, cardiac computed tomography and nuclear imaging are discussed with respect to atrial size, function, tissue characterization and substrate assessment, with particular emphasis on advanced imaging markers. Furthermore, pragmatic imaging-based algorithms are proposed for the evaluation and follow-up of AtCM in preclinical and overt heart failure, atrial fibrillation, cardiomyopathies, valvular heart disease, and peri-procedural settings. Knowledge gaps, unmet clinical needs and future research priorities are also highlighted. By integrating available evidence into a structured framework, this document aims to support a more standardized—yet personalized—approach to imaging-guided management of AtCM in clinical practice.

## Graphical Abstract

**Graphical Abstract:**
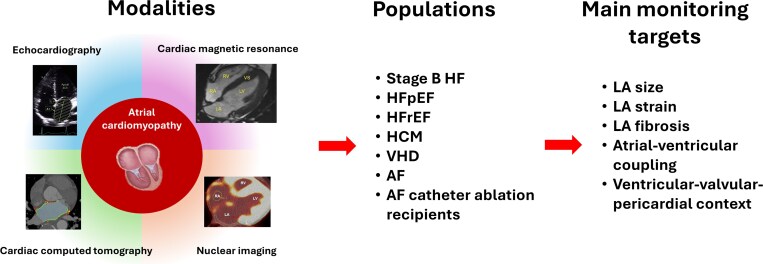

## Introduction

Atrial cardiomyopathy (AtCM) refers to a pathological atrial substrate characterized by atrial enlargement, impaired reservoir and contractile function, atrial fibrosis, electrophysiological abnormalities, and endothelial dysfunction. These structural and functional alterations predispose to adverse clinical outcomes, including atrial fibrillation (AF), heart failure (HF), and stroke.^1–6^ Atrial failure represents the advanced stage of AtCM, marked by progressive structural, functional, and electrical deterioration, accompanied by clinical symptoms.^7^

Early detection of subclinical atrial remodelling is essential for initiating timely therapeutic interventions and potentially reversing disease progression. However, clinical assessment alone often lacks the sensitivity required to identify early substrate changes or to monitor treatment response.^8^ Therefore, a structured, imaging-based surveillance strategy is essential. Multimodal cardiac imaging provides comprehensive insights into the extent and nature of atrial remodelling. It should be emphasized that, alongside imaging-derived markers of AtCM, the ECG offers an important diagnostic adjunct that enhances early disease recognition and contributes to clinical prognostication, although its role in longitudinal monitoring remains less well defined.

*Echocardiography* serves as the first-line imaging modality for diagnosing AtCM, delivering readily accessible and clinically relevant data on atrial size and function. In particular, atrial deformation analysis using speckle-tracking technique represents a significant advancement, improving the ability to detect and monitor early atrial impairment and to refine clinical risk stratification.^9,10^

*Cardiac magnetic resonance* (*CMR*) *with late gadolinium enhancement* (*LGE*) *and T1 mapping* remains the reference standard for quantifying atrial volumes and fibrosis. The extent of LGE–CMR burden, despite technical challenges in its measurement, is strongly associated with AF recurrence, procedural outcomes, and stroke risk.^3,11^

Additional imaging modalities—including *cardiac computed tomography (CCT)* for volumetric assessment, *nuclear imaging* (e.g. PET tracers targeting fibrosis or autonomic innervation), and advanced computational approaches such as biophysical simulations and machine learning—further enhance substrate characterization and risk evaluation.^12^

A recent Heart Failure Association of the ESC consensus statement established a contemporary clinical framework for AtCM, proposing updated definitions, diagnostic criteria, and staging based on electrical dysfunction combined with evidence of mechanical dysfunction, atrial enlargement, or fibrosis.^7^ The document identified imaging-based surveillance as a critical unmet need, noting the absence of standardized monitoring algorithms and the lack of modality-specific guidance across clinical scenarios.

This Scientific Statement focuses specifically on the role of imaging in monitoring AtCM and atrial failure. It presents the available evidence and seeks to: identify key knowledge gaps in imaging-based surveillance; review modality-specific strengths across echocardiography, CMR, CCT, and nuclear imaging; and propose practical imaging algorithms tailored to clinical scenarios associated with AtCM.

## Imaging techniques used for monitoring AtCM

**Figure 1:**
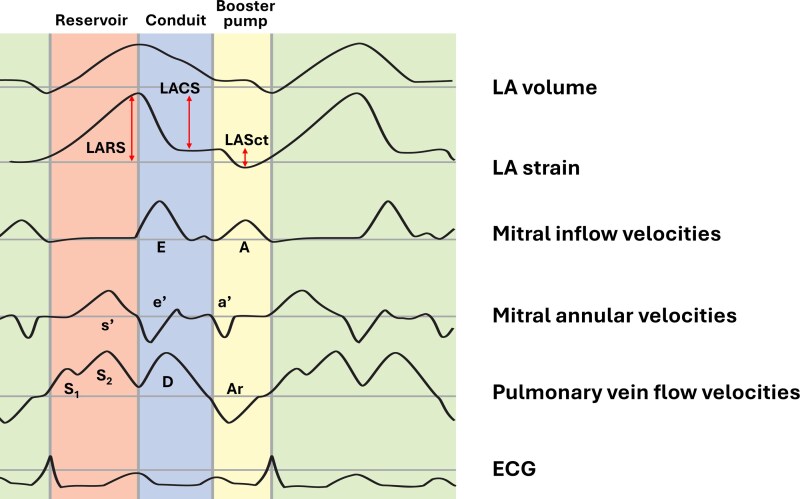
Phasic changes of atrial mechanics illustrated by curves derived from mitral inflow and pulmonary vein Doppler, tissue Doppler, volumetric analysis, and speckle-tracking techniques. A, late diastolic mitral inflow velocity; a′, late diastolic mitral annular velocity; Ar, pulmonary vein atrial reversal velocity; D, pulmonary vein diastolic velocity; E, early diastolic mitral inflow velocity; e′, early diastolic mitral annular velocity; LACS, left atrial conduit strain; LARS, left atrial reservoir strain; LASct, left atrial contractile strain; S_1_, S_2_, pulmonary vein systolic velocities; s′, systolic mitral annular velocity

Echocardiography is a scalable modality for diagnosing and tracking the progression of AtCM, due to its wide availability, clinical validity, and potential for automation.^13^ Left atrial (LA) size and volume, particularly LA volume indexed for body surface area (LAVi), have traditionally been used to identify adverse atrial remodelling, but echocardiography also enables assessment of atrial function.^14^ Phasic changes of atrial mechanics are captured by various parameters derived from mitral inflow and pulmonary vein Doppler, tissue Doppler, volumetric measurements and speckle-tracking techniques (*Figure 1*). Specifically, LA deformation analysis provides information on LA reservoir, conduit and booster pump function, offering important insights into cardiac pathophysiology.

Atrial remodelling markers are independently associated with incident or recurrent AF,^15,16^ HF,^17,18^ stroke,^19^ and cardiovascular mortality.^20,21^ Atrial dysfunction often represents an early sign of atrial remodelling,^22^ and LA strain abnormalities typically precede LA dilatation, offering diagnostic and prognostic value that is both independent of and incremental to LA size.^23–26^ Atrial morphological and functional markers reflect distinct pathomechanisms contributing to adverse atrial remodelling, such as atrial fibrosis or elevated cardiac filling pressures, and thereby mirror abnormalities in both atrial and ventricular physiology. Accordingly, atrial findings should be interpreted within a comprehensive framework that includes ventricular and valvular performance.^7^

Serial echocardiographic assessment can improve detection of both adverse and reverse atrial remodelling, particularly during targeted therapeutic interventions. Reversal of atrial remodelling is characterized by improved or restored atrial function or volume, although complete normalization is uncommon.^27^ Reverse atrial remodelling has been observed following rhythm control strategies in AF, as well as with medical and device therapies across various HF populations,^28–33^ and is associated with better outcomes even in the absence of ventricular reverse remodelling.^22,28–30^ As such, reverse atrial remodelling may serve not only as an indicator of treatment response but also as a prognostic biomarker for adverse outcomes, including incident AF or HF. Lack of improvement or progression of adverse remodelling may identify higher-risk patients^33^ and prompt reconsideration of therapeutic strategies, including interventions targeting alternative haemodynamic factors or underlying pathomechanisms.^7,27,32,34^

Data on serial atrial remodelling in patients at risk for, or with, AtCM—but without clinically evident AF or HF—remain scarce, although such information could enhance risk stratification and support earlier intervention targeting atrial pathologies.

Right atrial (RA) remodelling often parallels LA changes and is particularly relevant in pulmonary disease, pulmonary hypertension, or right ventricular/tricuspid valve dysfunction.^35–39^ A noteworthy aspect is biatrial myopathy, which may potentiate clinical and prognostic deterioration.^40^ Interest in imaging RA size and function is increasing,^41^ however clinical expertise remains limited, and echocardiographic assessment is frequently constrained by a suboptimal acoustic window.

**Figure 2:**
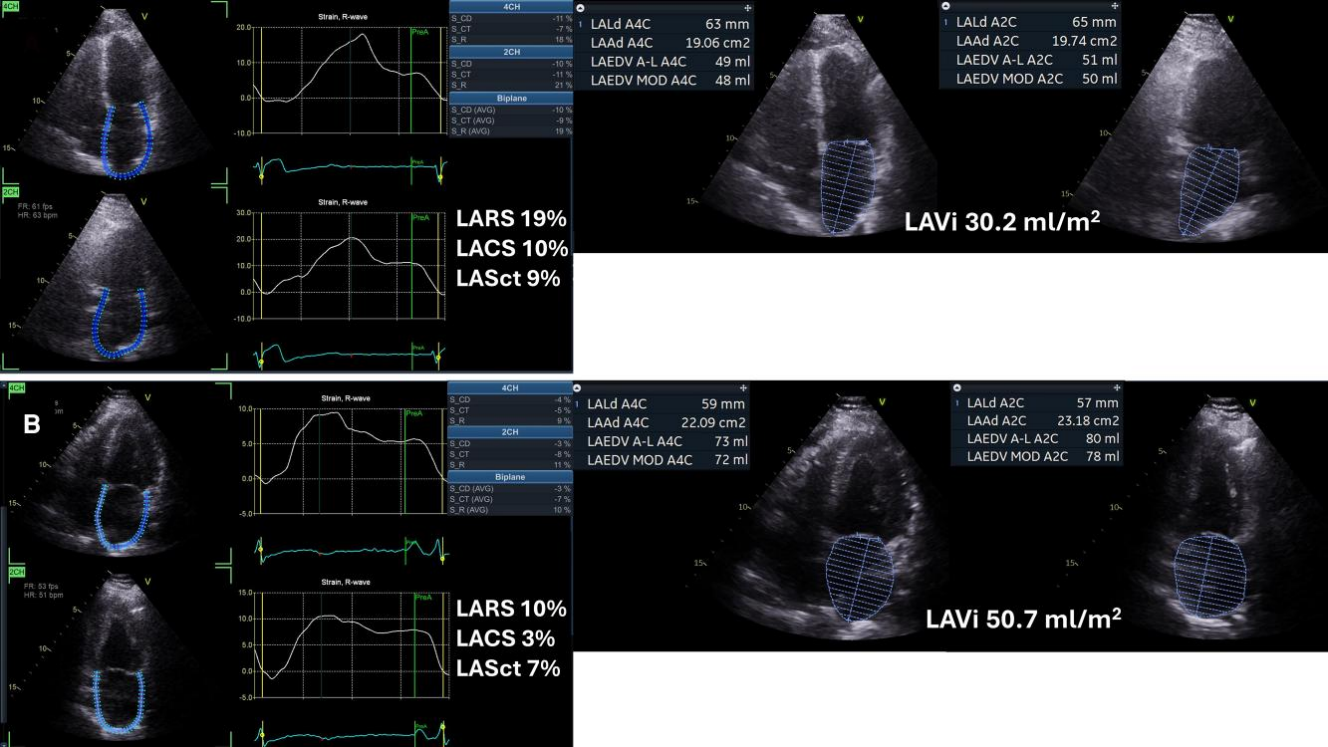
Different stages of atrial cardiomyopathy on echocardiographic imaging. Upper panel (*A*)—early AtCM with moderately reduced LA strain and normal LA volume. Lower panel (*B*)—advanced AtCM with severly reduced LA strain and LA dilatation, clinically consistent with atrial failure

Transthoracic echocardiography is the most practical tool for longitudinal monitoring (*Figure 2*). Volumetric measures, such as LAVi, show moderate reproducibility,^42^ whereas LA strain demonstrates excellent reproducibility when assessed using consistent software platforms.^34,43,44^ Three-dimensional (3D) echocardiography may improve the accuracy of LAVi measurement, but remains technically demanding.^34^ Transoesophageal echocardiography (TOE) is not well-suited for serial follow-up due to its semi-invasive nature, but it holds an important role in acute or pre-interventional decision-making, particularly when transthoracic imaging is suboptimal or inconclusive.

### Cardiac magnetic resonance imaging

CMR is the gold standard non-invasive imaging modality for quantification of cardiac chamber volumes due to its high spatial resolution and clear delineation of blood-endocardial borders. Reference values for LA^45,46^ and RA^45^ dimensions and volumes have been published. Steady-state free precession (SSFP) imaging enables continuous recording of phasic volumes throughout the cardiac cycle, allowing accurate quantification of atrial reservoir, conduit and contractile function. Beyond volumetric assessment, phasic LA and RA strain can be obtained using tissue-tracking CMR. Decreased CMR-derived LA strain has been observed in individuals with cardiovascular risk factors,^47^ and is associated with incident cardiovascular events in the general population.^48^

**Figure 3:**
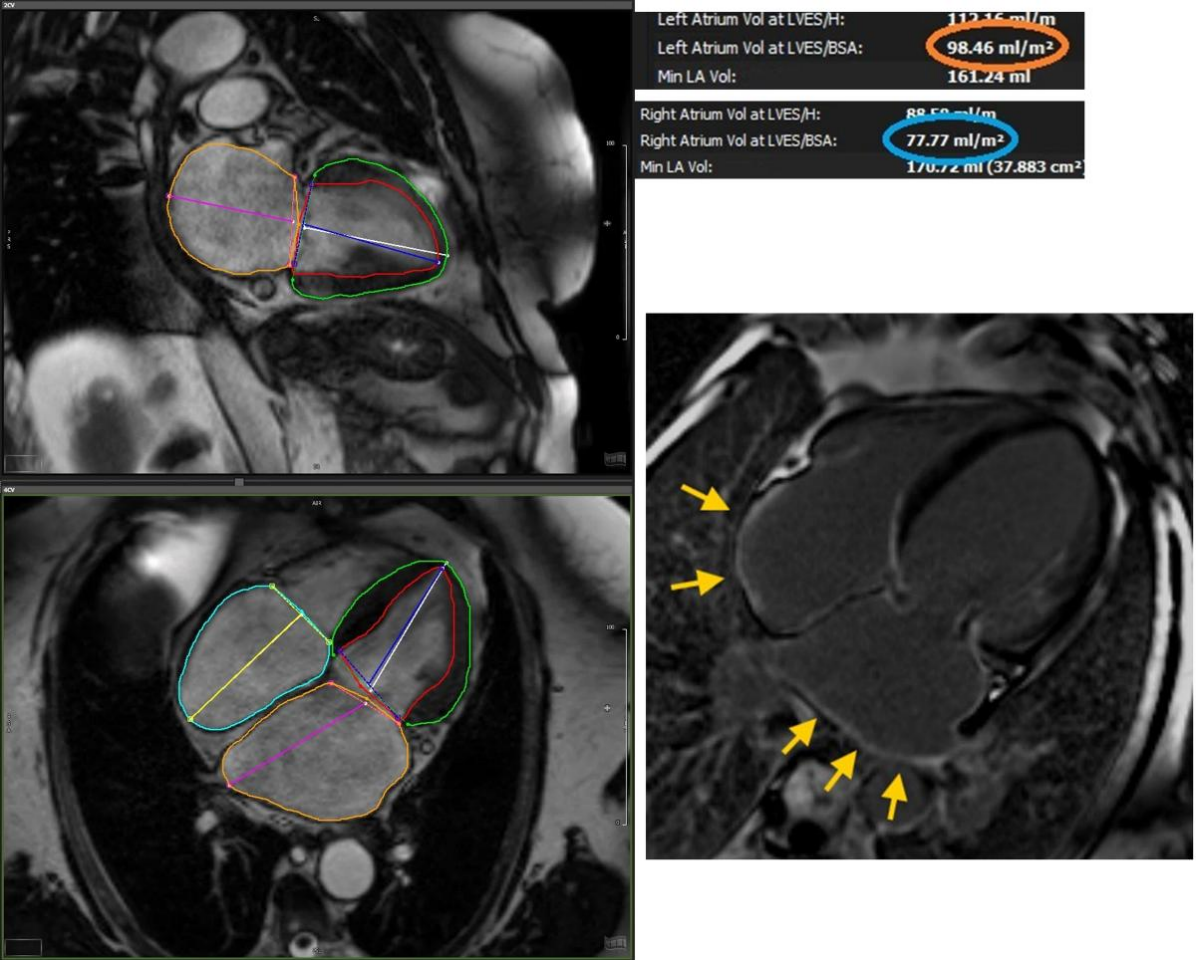
Features of biatrial cardiomyopathy on CMR. Left- and right-atrial dilatation and biatrial late gadolinium enhancement (arrows)

Atrial fibrosis is a hallmark of advanced AtCM,^32^ however histological assessment is rarely feasible in clinical practice.^49^ CMR offers a unique capability to detect a surrogate of regional atrial replacement fibrosis using late gadolinium enhancement (LGE) imaging (*Figure 3*).^3,50^ Atrial LGE has been shown to predict stroke risk in patients with^11,51^ and without^52,53^ AF and AF recurrence post-ablation,^54^ and provides mechanistic insights into AtCM across diverse conditions, including hypertrophic cardiomyopathy,^55^ mitral regurgitation,^56^ and in the general population.^57^ A 10–15% threshold of atrial wall enhancement on LGE-CMR is considered clinically meaningful for predicting outcomes in AF.^58^ To date, AF ablation strategies targeting atrial fibrosis have not improved rhythm outcomes,^59,60^ and atrial LGE assessment remains technically challenging, restricting its use to specialist centres. Further refinement and standardization of 3D-atrial LGE protocols are needed to enhance image quality and facilitate broader clinical adoption.

While 3D CMR imaging is routinely used to visualize the pulmonary veins and atrial anatomy for ablation planning and peri-procedural guidance,^61^ more sophisticated CMR assessment of the functional consequence of AtCM can be provided through intra-atrial flow dynamics. Abnormal atrial flow parameters, derived from 4D flow velocity mapping, have been linked to increased thromboembolic risk even in the absence of clinical AF.^62,63^ Emerging 5D flow mapping sequences have demonstrated improved resolution by accounting for both cardiac and respiratory motion.^64^

Further areas of development, supported by initial feasibility data, include T1 mapping of the LA for quantification of extracellular volume (ECV) fraction as a measure of diffuse atrial fibrosis,^65^ and black-blood CMR sequences for better visualization and evaluation of LA wall thickness.^66^ However, technical challenges and insufficient standardization still limit the applicability of these approaches in everyday clinical practice.

### Cardiac computed tomography

**Figure 4:**
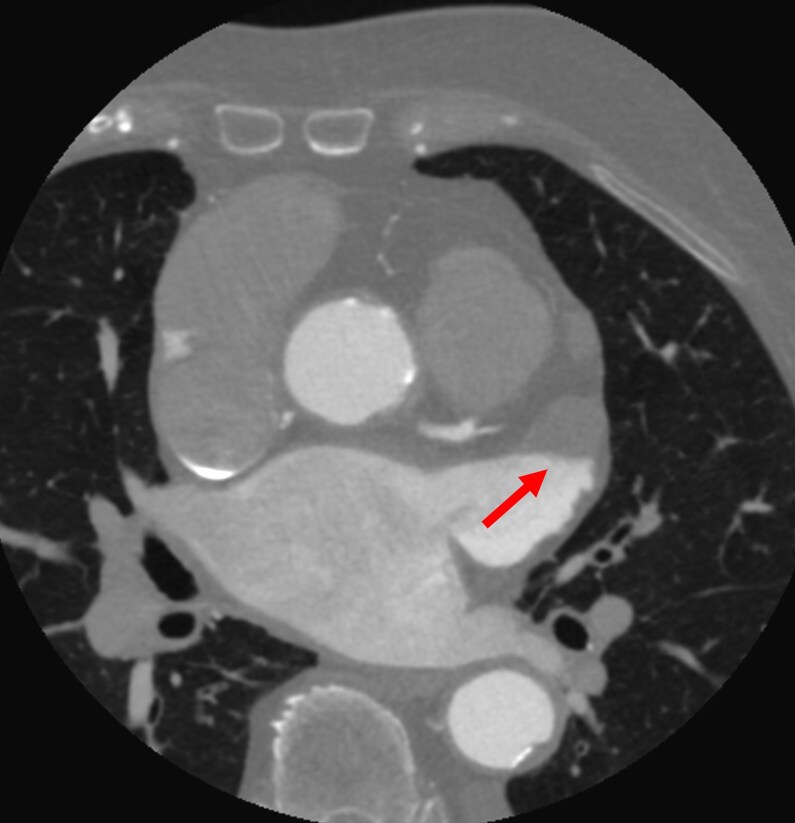
Thrombus at the bottom of left atrial appendage identified on computed tomography

CCT plays a complementary role in evaluating AtCM, particularly for structural assessment, thrombus exclusion, and pre-procedural planning, although its prognostic value in this condition remains to be fully elucidated (*Figure 4*).^67,68^ CCT provides high spatial resolution for quantifying LA and RA volumes, chamber geometry, and wall thickness, all of which are relevant to atrial remodelling and characterization of the arrhythmogenic substrate.

**Figure 5:**
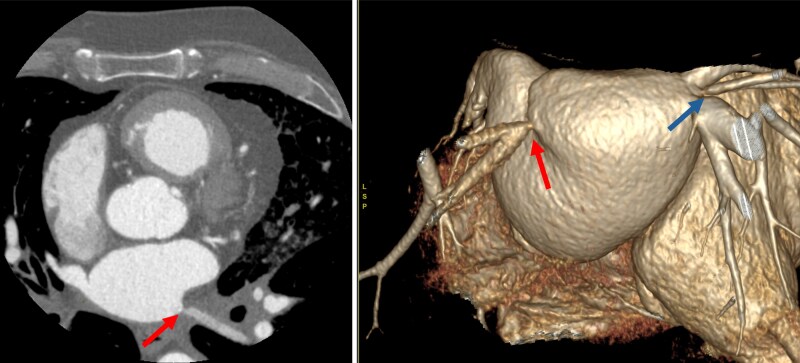
CCT imaging of post-ablation pulmonary vein (PV) stenosis. Left panel: axial view; right panel: 3D reconstruction. The red arrow indicates ostial stenosis of the left inferior PV, and the bluearrow indicates ostial stenosis of a right accessory PV. Note the absence of the totally occluded left superior PV on 3D imaging

Pre-ablation LA volume and chamber asymmetry on CCT have been shown to predict sinus rhythm maintenance after ablation procedures.^49^ CCT is increasingly used to exclude LA appendage thrombus, particularly in patients undergoing cardioversion or catheter ablation for AF, with reported sensitivity and specificity of 96% and 92%, respectively, improving to 99% and 100% with delayed imaging protocols. The high negative predictive value, approaching 99%, supports its integration into routine pre-ablation workflows.^69^ CCT provides accurate delineation of pulmonary vein anatomy and anatomical variants, which is essential for AF ablation and LA appendage closure planning,^67,70^ and it also helps identify procedural complications (*Figure 5*).

Recent studies have expanded the role of CCT to include quantification of atrial wall thickness and epicardial adipose tissue. Patients with AF recurrence have been shown to exhibit significantly larger indexed LA volumes, increased anterior LA wall thickness, higher epicardial adipose tissue volume, and lower epicardial fat attenuation, indicating increased lipid content.^71^ The addition of LA wall thickness to established risk models has demonstrated significant incremental prognostic value.

CCT-based assessment of atrial remodelling and fibrosis is evolving, supported by advanced post-processing, computational modelling, and machine-learning approaches for personalized risk stratification. Its rapid acquisition and high spatial resolution make CCT particularly useful in patients with suboptimal echocardiographic windows or contraindications to CMR.

### Nuclear imaging

Nuclear imaging of the atria has advanced rapidly, with positron emission tomography (PET) using dedicated tracers—^18^F-fluorodeoxyglucose (FDG) and radiolabeled fibroblast activation protein inhibitors (FAPI)—which enable *in vivo* assessment of atrial inflammation and fibrosis. However, PET markers for AtCM are still in the investigational stage.

FDG-PET, when performed with optimized glucose suppression protocols, can reliably detect atrial hypermetabolism as a marker of inflammation in AF.^72,73^ Quantitative FDG-PET analysis has demonstrated sensitivity and specificity for identifying AF of 65.7% and 75.8%, respectively, with higher RA target-to-background ratios in persistent AF and histopathological confirmation of inflammatory cell infiltration in regions of increased uptake.^72,73^ Enhanced atrial FDG activity is also independently associated with AtCM and prior ischaemic stroke, even in individuals without AF.^74^ Advances such as digital time-of-flight PET have further improved quantitative evaluation of LA metabolism.^75^

FAPI-PET represents a novel approach for imaging atrial fibrosis and fibroblast activation. In a proof-of-concept study, this technique identified enhanced atrial fibroblast activity in 64.3% of AF patients, with no uptake observed in healthy controls.^76^ Atrial FAPI activity showed strong correlations with FAP mRNA expression (*r* = 0.98), FAP protein expression (*r* = 0.82), and collagen I mRNA (*r* = 0.85). Elevated B-type natriuretic peptide levels were associated with increased atrial FAPI uptake, supporting the link between fibroblast activation and atrial remodelling.

Beyond FDG and FAPI, several additional tracers are under investigation for AtCM monitoring. Somatostatin receptor–targeted agents (e.g. ^68^Ga-DOTATOC) show feasibility for imaging cardiac inflammation with potentially improved specificity over FDG, although their application in atrial imaging is still investigational.^77^ Cellular proliferation tracers, such as ^18^F-fluorothymidine (^18^F-FLT), target DNA synthesis in proliferating cells and have shown promise for imaging cardiac inflammation with potentially lower physiologic myocardial uptake than FDG.^78,79^ Amyloid-targeted tracers, including ^99m^Tc-pyrophosphate and ^18^F-labeled agents (florbetapir, flutemetamol, florbetaben), are well established for cardiac amyloidosis and may help detect atrial amyloid infiltration, which contributes to atrial remodelling and arrhythmogenesis.^79,80^

### Key points

Imaging techniques for AtCM monitoring

- Atrial remodelling markers (structural and functional) may reflect disease progression, treatment response, and prognosis.
- Evidence remains predominantly LA-focused; data on RA remodelling and serial changes—especially in early AtCM—are still limited.
- Echocardiography is the first-line tool for AtCM surveillance, providing information on atrial size and function. STE-derived strain (reservoir, conduit, booster pump) enables early detection and longitudinal monitoring, offering incremental value beyond chamber size; 3D echo improves volumetric accuracy.
- CMR is the reference for atrial volumetry and phasic function (including strain) and detects replacement fibrosis via LGE. 3D CMR enhances anatomical assessment, while emerging methods (intra-atrial flow, ECV mapping, black-blood sequences) may refine substrate characterization.
- CCT provides complementary structural evaluation, supports thrombus exclusion and procedural planning, and can quantify atrial wall thickness and epicardial adipose tissue.
- Nuclear imaging (FDG PET for inflammation, FAPI PET for fibrosis/fibroblast activity, amyloid-targeted tracers) offers additional insights into atrial substrate pathology.

## Monitoring AtCM in heart failure and specific aetiologies of myocardial impairment

### Stage B heart failure and lifestyle interventions

In contemporary assessments of stage B heart failure (SBHF), defined by evidence of structural heart disease or dysfunction in the absence of HF signs or symptoms, atrial markers have gained increasing attention, reflecting recognition that LA remodelling may serve as a more integrative indicator of subclinical myocardial dysfunction than ventricular changes.^7,81^ Alterations in LA size and mechanics, including volume, strain, and stiffness parameters, across the spectrum of HF severity collectively delineate the trajectory of atrial substrate remodelling, linking intrinsic atrial pathology with adaptive responses to elevated filling pressures.^7^

In hypertensive heart disease, early alterations in LA mechanical function during exercise precede symptomatic HF and predict clinical outcomes.^82^ The LA reservoir strain response to exercise progressively deteriorates from stage A through stage B to stage C, correlating independently with exercise capacity but not reliably distinguishing symptomatic from asymptomatic individuals. In contrast, the LA stiffness response to exercise—derived from LA strain and the E/e′ ratio remains stable in preclinical HF and rises with the advent of HF symptoms. LA strain reserve provides incremental prognostic value for HF worsening and new-onset AF.^82^ These findings suggest that dynamic changes in LA strain-derived indices during exercise reflect subclinical atrial–ventricular interaction and help identify individuals at elevated risk of functional decline and transition to overt HF. The contribution of reduced LA functional reserve in the development of impaired exercise capacity has been confirmed in another study in preclinical HF.^83^ Complementary CMR indices, such as LA volumetry and emerging techniques for atrial tissue characterization, further support this atrial-centred approach to risk stratification, although the clinical application of LA T1 mapping remains investigational.^47,65,84^

In the community-based MESA cohort, more advanced LA remodelling on CMR—larger maximal/minimal volumes, reduced strain, and lower emptying fraction—predicted incident HF and cardiovascular disease.^85,86^ Declining peak LA strain and rising pre-atrial contraction volume over time were associated with HFpEF and HFrEF development, respectively.^87^

In individuals with hypertension, diabetes, metabolic disease or prior exposure to cardiotoxic therapies, atrial dysfunction may represent the earliest measurable manifestation of myocardial impairment. Lifestyle modification is an essential component of AF prevention and SBHF management, particularly when early atrial dysfunction is identified.^88^ Targeted interventions, including structured aerobic exercise, weight reduction, and improved metabolic control, have been shown to favourably influence diastolic function and determinants of atrial remodelling.^89,90^ Aerobic training and weight loss can improve ventricular relaxation and LA strain metrics, thereby mitigating progression toward myocardial stiffness and HF.^88–90^

### Key points

Stage B HF

- LA remodelling may serve as integrative indicator of subclinical myocardial dysfunction.
- LA volumetric and deformation markers predict the risk of HF development.
- Early alterations in LA mechanical function during exercise precede symptomatic HF and predict clinical outcomes.
- Lifestyle interventions, including aerobic exercise, weight reduction, and improved metabolic control, ameliorate atrial remodelling and slow progression toward HF.

### HFpEF

#### Pathophysiologic insights

Contrary to the previous belief that LA dilatation in HFpEF is a late outcome of chronically elevated filling pressures,^91,92^ contemporary data show early LA structural and functional remodelling in this condition.^93^ This supports a paradigm of global cardiac remodelling in HFpEF pathogenesis.^94^ Pathologies originating in the atrial myocardium are further promoted by the haemodynamic consequences of impaired LV filling, resulting in LA dilatation, increased LA stiffness, and contractile dysfunction, with atrial fibrosis and myocardial degeneration on histopathology.^93,95^ Maladaptive left atrioventricular coupling also develops and adversely affects LV systolic performance.^93,95^ AF is common in HFpEF and both arises from and accelerates underlying AtCM.^96^

#### Imaging AtCM to support HFpEF diagnosis and phenotyping

Diagnostic algorithms for HFpEF include echocardiographic assessment of LA volume index (LAVI), with thresholds >34 mL/m^2^ (>40 mL/m^2^ in AF) considered a major distinguishing criterion, reflecting chronic LA hypertension.^97^ LA reservoir strain <18–19% can be useful for identifying elevated LV filling pressures, thereby improving diagnostic sensitivity.^98–100^ CMR-derived minimal LA volume and emptying fraction also carry diagnostic value in this context,^101^ while LA roof dilatation on CMR may precede LA enlargement, unmask early-stage (exercise-induced) HFpEF, and predict AF.^102^

Heterogeneous AtCM patterns have been observed in HFpEF, including primary (disproportionate) LA remodelling, isolated RA remodelling (rare), and biatrial myopathy, the latter reflecting more advanced disease.^40^

#### Imaging AtCM to monitor HFpEF progression

Combined volumetric and strain assessment of both atria can enhance risk stratification in HFpEF. LA mechanical dysfunction correlates with NYHA class,^95^ while CMR-derived LA strain is associated with exercise intolerance.^103^ Echocardiographic LA reservoir strain predicts outcomes more effectively than LV GLS or RV free-wall strain and supports AF risk stratification.^25,104^ The value of LA functional parameters—LA reservoir strain measured both at rest and during exercise, and LA emptying fraction—for predicting adverse events in HFpEF has been confirmed irrespective of AF status.^21,105,106^ Impaired LA functional reserve, reflected by abnormal exercise-induced changes in strain and stiffness, may represent the earliest sign of LA failure, heralding the onset of symptomatic HFpEF.^82^

The dynamic interaction between the LA and LV contributes to atrial remodelling, playing a pivotal role in HF progression and informing prognosis. Although this pathophysiological interplay is evident across HF categories, it is particularly relevant in HFpEF. The left atrioventricular coupling index (LACI), defined as the LA/LV volume ratio at end-diastole, predicts mortality as well as HF and CV hospitalizations.^105,107^ Other indices of atrial–ventricular interaction—including the LA filling index (calculated as the ratio of mitral early-diastolic inflow peak velocity (E) to LA reservoir strain),^108^ the LA strain to E/e′ ratio,^109^ and the LAVi to late diastolic mitral annular velocity (a′) ratio^110^—are likewise predictive of adverse outcomes and elevated LV filling pressures in HFpEF.

Data on the potential of RA markers to stratify clinical risk in HFpEF are less extensive. However, existing evidence suggests that RA reservoir and conduit strains may have predictive value for mortality and HF hospitalization, particularly in sinus rhythm.^111,112^

#### Imaging AtCM to monitor treatment response in HFpEF

Data on the use of imaging to monitor AtCM as a marker of treatment response in HFpEF remain scarce. Serial evaluation of atrial volumes and function following atrial septal shunt implantation has shown reductions in minimal LA volume and increases in RA volume, paralleling improvements in haemodynamic parameters and quality of life in selected patients with HFpEF.^113^

Advanced AtCM can lead to the development of atrial functional mitral regurgitation, worsening pulmonary hypertension and eventual or concomitant tricuspid regurgitation (TR) with RA remodelling. Recent evidence suggests that the development of TR may not be solely a late secondary consequence of LV diastolic dysfunction, but can emerge in earlier stages of HFpEF in response to RA remodelling, which—along with left heart abnormalities—is driven by systemic pro-inflammatory and pro-fibrotic stimuli.^114^ randomized clinical trials are needed to confirm observational findings that mitral or tricuspid valve intervention can reverse AtCM and improve outcomes in HFpEF.^115^

An overview of evidence on AtCM biomarkers in HFpEF is presented in *Table 1*.

**Table 1:** Selected evidence on AtCM markers in HFpEF

Baseline evaluation of AtCM in HFpEF				
Echocardiography	Parameters	Cut-off	Clinical relevance	Limitations and gap in evidence
Atrial size as a part of diagnostic algorithm for HFpEF	LAVi		Left atrial geometric remodelling; left ventricular filling pressure	Two-dimensional evaluation may underestimate volumes
	In sinus rhythm^95,116,117^	34 mL/m^2^	Upper limit of normative ranges, predictive of death, HF, AF and stroke	
	In AF^118^	40 mL/m^2^	LAVi 35% larger in HFpEF with AF versus sinus rhythm	Threshold defined on the basis of expert opinion
Atrial strain	LARS/LASct		Functional remodelling which precedes geometric remodelling; identification of LV filling pressure elevation Prediction of HF worsening Prediction of AF occurrence	Wide range of proposed cut-off values Limited data in permanent AF patients Uncertain applicability in AF, significant mitral regurgitation, heart transplant recipients
		LARS <18%^119^	Accuracy 72% for detecting LV filling pressure elevation in preserved LVEF	
		LASct <14%^119^	Accuracy 92% for detecting LV filling pressure elevation when GLS >18%	
		LARS <22.7%^21^	Increased risk of HF hospitalization and overall mortality independent of AF presence	
		LARS <29.4% and LASct <12.7%^25^	Used together with LAVI >34.3 mL/m^2^ to predict incident AF	
		LARS <31.2%^104^	Increased risk of HF hospitalization, CV hospitalization, or death	
		LARS <27.8% (median)^105^	Increased risk of CV hospitalization and mortality irrespective of AF presence	
		LARS (no cut-point identified)^120^	1-unit (1%) decrease associated with 5% higher hazard of HF hospitalization	
		LARS <19.3% (AF) or 20.8% (SR)^100^	Specificity 86% for diagnosing HFpEF versus symptomatic controls when combined with natriuretic peptides	
AV coupling and LV filling pressure	LACI	≥0.26^117^	Identification of ≥moderate LV diastolic dysfunction (AUC 0.75)	Limited data about the reproducibility of measurements
		≥0.32^107^	Prediction of all-cause death or HF hospitalization	Not standardized cut-offs
		≥0.235^121^	Identification of ≥moderate LV diastolic dysfunction (AUC 0.74)	
		≥0.26^121^	Prediction of all-cause mortality, HF hospitalization or progression to end-stage kidney disease	
	LAVi/a’	≥6.0^110^	Prediction of CV death or HF hospitalization	
	E/LARS	>3.27^108^	Identification of LV filling pressure elevation (AUC 0.82; sensitivity 83.3%, specificity 78.9%) Prediction of HF hospitalization	
	LARS/E/e′	<2.1^a109^	Identification of LV filling pressure elevation (AUC 0.83; sensitivity 88%, specificity 74%)	

CMR	Parameters	Cut-off	Clinical relevance	Limitations and gap in evidence
Atrial parameters in diagnosing LV filling pressure elevation	Minimal LA volume^101^	23 mL/m^2^	Sensitivity 86%; Specificity 63% for detecting LV end-diastolic pressure elevation	Limited data No clear cut-off values Expensive and time consuming approach
	LA emptying fraction^101^	35%	Sensitivity 71%; Specificity 89% for detecting LV end-diastolic pressure elevation	
Atrial function	LA emptying fraction^106^	No specific cut-point identified	Prediction of all-cause death or HF hospitalization (HR 0.767, 95% CI 0.591–0.996)	
	LA reservoir strain^105^	<30.5% (median)	Increased risk of CV hospitalization and mortality irrespective of AF presence	
	RA conduit strain (mixed population; 48% HFpEF)^112^	No specific cut-point identified	Prediction of all-cause mortality (HR 0.58; 95% CI: 0.40 to 0.84)	
	RA reservoir and conduit strain^111^	No specific cut-points identified	Prediction of HF hospitalization or CV death in patients with sinus rhythm but not with AF	
AV coupling	LACI at rest and exercise^122^	No cutpoints provided ≥36.93% at rest and ≥34.23% at exercise (median values)	Identification of LV filling pressure elevation (AUC rest 0.81; stress 0.83) Prediction of CV hospitalization	

AF, atrial fibrillation; AtCM, atrial cardiomyopathy; AV, atrioventricular; CV, cardiovascular; E, peak early diastolic mitral inflow velocity; e′, peak early diastolic mitral annular velocity; a′, peak late diastolic mitral annular velocity; LARS, left atrial reservoir strain; LASct, left atrial contractile strain; HF, heart failure; HFpEF, heart failure with preserved ejection fraction; LACI, left atrial coupling index; LAVi, maximum left atrial volume indexed for body surface area; LV, left ventricular; LA, left atrial; RA, right atrial; SR, sinus rhythm.

^a^Cut-off also varying on the basis of e′ calculation (septal, lateral or mean of septal and lateral values).

### Key points

HFpEF

- Atrial remodelling may reflect early atrial myocardial pathology as well as later consequences of elevated LV filling pressures, and, synergizing with commonly coexisting AF, contributes to disease progression.
- Atrial volumetric and functional (strain) measures improve prediction of mortality, HF worsening, AF occurrence, and stroke.
- LA dysfunction correlates with exercise intolerance.
- LA strain provides stronger prognostic information than LV and RV strain.
- LA–LV coupling indices predict adverse outcomes and help identify elevated LV filling pressures.
- RA strain offers additional prognostic value for mortality and HF hospitalization

### HFrEF

#### Diagnostic algorithm in HFrEF

The pathophysiological milieu linking AtCM and heart failure with reduced ejection fraction (HFrEF) encompasses disease-specific factors, such as genetic cardiomyopathies, myocarditis, and ischaemic heart disease, as well as elevated LV filling pressure and AF, all of which contribute to atrial remodelling.^32,123,124^ This interaction creates a vicious cycle that accelerates HF progression,^123,125^ underscoring the need for accurate assessment of AtCM-related parameters.

Baseline echocardiographic assessment of LA morphology and function—particularly LA volume and strain—provides diagnostic and prognostic information and helps predict mortality, HF progression, and AF development.^126–132^ LA strain has been shown to be prognostically superior to both LV GLS and LAVI in HFrEF.^128^ RA volume and strain likewise carry prognostic value in this population.^133–135^ As in HFpEF, parameters reflecting LA–LV coupling- such as LACI,^107,136^ the ratio of LAVi to a′,^137^ the LA filling index, and the ratio of LA strain to E/e′^130^—are consistently associated with unfavourable outcomes in this HF category.

CMR further refines prognostic assessment in HFrEF. LA reservoir strain and atrial fibrosis on LGE provide independent and incremental risk stratification for major cardiovascular events, including cardiovascular death, stroke, and thromboembolism.^125^ CMR-derived RA geometric and functional abnormalities also predict adverse prognosis.^112^

#### Monitoring AtCM in HFrEF

LA reverse remodelling can serve as a useful marker of therapeutic response in HFrEF. Angiotensin receptor–neprilysin inhibitors (ARNIs) have demonstrated superiority over ACE inhibitors and angiotensin II receptor blockers in attenuating LA dilatation,^138^ with improved outcomes in patients with mildly enlarged LA size. Likewise, significant reversal of LA remodelling has been reported with type 2 sodium–glucose cotransporter (SGLT2) inhibition.^139^ After acute decompensation, treatment-related changes in LA volume and strain may help track decongestion and act as early surrogate markers for long-term outcomes.^132,140^

Selected evidence on AtCM biomarkers in HFrEF is summarized in *Table 2*.

**Table 2:** Selected evidence on AtCM markers in HFrEF

Baseline evaluation of AtCM in HFrEF				
Echocardiography	Parameters	Cut-off	Clinical relevance	Limitations and gap in evidence
Atrial size	LAVi^116^	34 mL/m^2^	Left and right atrial geometric remodelling	Two-dimensional evaluation may underestimate volumes
	RAVi^133^	32 mL/m^2^	Left and right ventricular filling pressure	AtCM may be present in the absence of geometric remodelling
Atrial strain	LARS^126–132,141^	Ranged between <12% and 23%	Functional remodelling which precedes geometric remodelling Prediction of mortality and HF worsening Prediction of AF occurrence	Wide range of proposed cut-off values No data in permanent AF patients
	LASct^130^	<10.0%	Prediction of HF progression (HF hospitalization, heart transplantation or CV death)	
AV coupling and LV filling pressure	LACI	≥0.32^107^ ≥30.9%^136^	Prediction of all-cause death or HF hospitalization Prediction of all-cause death or HF hospitalization	Limited data about the reproducibility of measurements Not standardized cut-offs
	LAVi/a′	≥6.0^137^	Prediction of overall mortality	
	E/LARS LARS/E/e′	>3.7^130^ <1.27^130^	Prediction of HF progression (HF hospitalization, heart transplantation or CV death)	

CMR	Parameters	Cut-off	Clinical relevance	Limitations and gap in evidence
Atrial size	LAVi		Atrial geometric remodelling Prediction of MACE and HF progression	Limited data Expensive and time consuming approach
Atrial strain	LARS	<9.1% in ischaemic aetiology;	Atrial functional remodelling	
		<13.75% in non-ischaemic aetiology^125^	Prediction of mortality, stroke, heart transplantation or HF hospitalization	

Serial evaluation of AtCM in HFrEF				
Echocardiography	Parameters	Cut-off	Clinical relevance	Limitations and gap in evidence
Atrial reverse remodelling	LAVi reduction^17,138,139^	>15%	Better HFrEF prognosis Therapeutic target	Arbitrary cut-offs Limited data about the role as therapeutic target
	LARS improvement^132,140,142^	>5%	Response to therapeutic strategies such as ARNi and SGLT2i Prognostic value after ADHF	

ADHF, acute decompensated heart failure; AF, atrial fibrillation; ARNi, Angiotensin Receptor neprylisin inhibitors; AtCM, atrial cardiomyopathy; AV, atrioventricular; CV, cardiovascular; E, peak early diastolic mitral inflow velocity; e′, peak early diastolic mitral annular velocity; a′, peak late diastolic mitral annular velocity; LARS, left atrial reservoir strain; LASct, left atrial contractile strain; HF, heart failure; HFrEF, heart failure with reduced ejection fraction; LACI, left atrial coupling index; LAVi, maximum left atrial volume indexed for body surface area; MACE, major adverse cardiovascular events; RAVi, maximum right atrial volume indexed for body surface area; SGLT2i, inhibitors of sodium glucose cotransporter.

### Key points

HFrEF

- LA structural and functional remodelling predicts adverse outcomes, including mortality, HF progression and AF development.
- LA strain prognostically outperforms LVGLS and LAVI, and together with LA LGE provides incremental prognostic value.
- LA-LV coupling indices are associated with unfavourable outcomes.
- ARNIs and SGLT2 inhibitors improve atrial remodelling, and LA volume and strain may help track decongestion.

### Hypertrophic cardiomyopathy

#### Sarcomeric hypertrophic cardiomyopathy

AtCM is an integral feature of hypertrophic cardiomyopathy (HCM), acting as both a marker of disease severity and a predictor of adverse cardiovascular events. It contributes substantially to the development of AF—the most common arrhythmia in HCM—with a prevalence of 20–25% across cohorts.^143,144^ The pathophysiology of AtCM in HCM is multifactorial and includes impaired diastolic filling of a thickened and non-compliant LV; mitral regurgitation resulting from systolic anterior motion (SAM) of the mitral valve leaflets; and genetic predispositions that promote atrial fibrosis and remodelling, such as sarcomeric mutations in the MYBPC3 and MYH7 genes, or the 344T > C polymorphism in the CYP11B2 gene.^145,146^

AF onset in HCM represents a major inflection point, as atrial contraction is crucial for LV filling in these patients and its loss is therefore particularly deleterious. Consequently, AF is often poorly tolerated and is associated with increased mortality, HF hospitalization, stroke, and reduced quality of life.^143,147^

Predicting the emergence of AF in HCM remains challenging. However, early structural and mechanical remodelling of the LA can be detected with imaging. LA size independently predicts adverse outcomes, including AF and thromboembolic stroke.^146^ A LAVi >34 mL/m^2^ is associated with higher risk of mortality, heart transplantation, sudden arrhythmic death, and ICD implantation.^148^ In recognition of LA enlargement as a strong predictor of sudden cardiac death, LA anteroposterior diameter has been incorporated into the HCM-SCD Risk Score.

Recent data indicate that reduced LA reservoir strain—typically below 18 to 23.4%—reliably predicts new-onset AF in HCM, independent of LA size.^149–151^ Its ability to identify AF risk and other adverse events supports its potential inclusion into prognostic tools to refine individualized treatment strategies.^149,150,152^

Beyond volumetric and functional indices, patients with HCM and AF exhibit a greater burden of LA myocardial fibrosis than those in sinus rhythm, suggesting LA fibrosis as an additional imaging biomarker for AF prediction.^153^

### Key points

HCM

- AtCM is an integral component of HCM and contributes substantially to AF, which increases mortality, HF hospitalization, stroke risk, and reduces quality of life.
- LA enlargement independently predicts adverse outcomes, including AF, stroke, and sudden cardiac death.
- Reduced LA reservoir strain (<18%–23.4%) predicts incident AF independent of LA size.
- LA fibrosis by CMR may provide an additional imaging marker for AF prediction.

#### Cardiac amyloidosis

The background of atrial impairment in cardiac amyloidosis is shaped predominantly by the primary infiltration of atrial myocardial tissue, with haemodynamic consequences of LV diastolic dysfunction playing a secondary role. Both transthyretin-related (ATTR) and light-chain (AL) amyloidosis feature progressive interstitial deposition of amyloid fibrils within the atrial myocardium, accompanied by fibrosis, leading to increased stiffness, reduced compliance, and electromechanical uncoupling.^154^

Imaging plays a central role in detecting and monitoring amyloidosis-related AtCM. Standard echocardiographic measures such as atrial volume have limited sensitivity for early disease, whereas atrial strain—particularly in the reservoir and contractile phases—offers incremental diagnostic value.^34^ As in other cardiac conditions, strain impairment typically precedes atrial enlargement and independently predicts adverse outcomes, highlighting its potential as a non-invasive marker of disease burden.^155^

CMR further improves diagnostic accuracy through detailed tissue characterization, permitting detection of diffuse atrial interstitial expansion, with LGE revealing circumferential or patchy enhancement suggestive of amyloid infiltration.^156^

The significance of the primary infiltrative component of AtCM in amyloidosis is highlighted by recent data in ATTR, where LA strain and LA stiffness showed weak correlation with LV diastolic dysfunction, yet remained predictive of thromboembolic events independent of CHA_2_DS_2_-VASc score and AF.^157^ These findings have important implications for anticoagulation strategies, arrhythmic risk profiling, and therapeutic monitoring in cardiac amyloidosis.

### Key points

Cardiac amyloidosis

- Primary atrial myocardial infiltration is a dominant driver of AtCM in amyloidosis.
- LA reservoir strain may outperform volumetric indices in detecting early atrial impairment and refining clinical risk prediction.
- LA strain and stiffness predict thromboembolic events independent of CHA_2_DS_2_-VASc score and AF, and their limited association with LV diastolic dysfunction underscores the central role of primary atrial infiltration.

### Valvular heart disease

Valvular heart disease (VHD), particularly involving the mitral and tricuspid valves, underpins the development of AtCM and atrial failure, primarily through chronic atrial pressure or volume overload. Resulting structural and functional atrial remodelling contributes to AF, thromboembolism, and HF symptoms.^158^

Imaging, particularly echocardiography with strain analysis and CMR with fibrosis assessment, plays a pivotal role in the comprehensive evaluation of atrial pathology in VHD. Imaging-derived markers of AtCM have direct implications for risk stratification, timing of valve intervention, and rhythm management. Reduced LA reservoir strain (<30%) has been independently associated with progression to symptomatic mitral regurgitation (MR) or aortic stenosis (AS), as well as an increased incidence of HF events.^159^ After mitral valve repair, decreased LA strain has emerged as a strong predictor of cardiovascular mortality, HF hospitalization, and reoperation during mid-term follow-up.^160–162^ In both AS and MR, myocardial fibrosis—whether reactive or replacement—has been linked to suboptimal reverse remodelling after valve surgery. ECV quantification has been independently predictive of mortality following aortic valve intervention.^163^

Serial assessments of LA strain and volumetric parameters have proven valuable in monitoring therapeutic response and identifying candidates for valve repair strategies, such as MitraClip,^164,165^ and transcatheter tricuspid interventions.^166^ Integrating advanced imaging into routine clinical evaluation may enhance risk assessment, inform anticoagulation and other treatment strategies, and support personalized therapeutic decision-making in VHD.

### Key points

Valvular heart disease

- Imaging-derived markers of AtCM inform risk stratification, timing of valve intervention, and rhythm management.
- LA reservoir strain <30% is independently associated with progression to symptomatic MR or AS.
- Reduced LA strain strongly predicts death, HF hospitalization, and reoperation after mitral valve repair.
- Serial assessments of LA strain and volumetric parameters help monitor therapeutic response and identify candidates for transcatheter mitral and tricuspid valve repair.

## Monitoring AtCM in atrial fibrillation: prediction of thromboembolic risk

Cardiovascular imaging plays a critical role in an integrated approach to AF management promoted by the 2024 European Society of Cardiology (ESC) guidelines —particularly in initial evaluation, rhythm control decision-making, and refined thromboembolic risk prediction.^2,167^ Echocardiography remains essential for assessing cardiac structure and function at diagnosis and throughout follow-up, while CCT and CMR are increasingly used to characterize specific cardiac conditions and guide peri-procedural planning.

### Transthoracic echocardiography at AF diagnosis: substrate characterization

TTE is the initial imaging modality of choice in nearly all patients with newly diagnosed AF that aids in symptom assessment and pharmacologic management guidance, particularly in selecting rate or rhythm control strategies and determining anticoagulation needs.^2,167^

Substantial evidence supports LA enlargement, in particular maximum LA volume index, as a robust marker of AF chronicity and thromboembolic risk.^168^ Additionally, impaired LA compliance has been linked to AF onset and its progression toward a persistent AF phenotype.^169^ LA strain imaging offers further insights by quantifying atrial functional remodelling, progressively recognized as independent predictor of stroke and arrhythmia recurrence.^170^

### Transoesophageal echocardiography: thromboembolic risk and procedural safety

Transoesophageal Echocardiography (TOE) plays a crucial role in detecting left atrial appendage (LAA) thrombus. Due to its high sensitivity for identifying thrombus, spontaneous echo contrast (SEC) and LAA dysfunction, TOE may provide additional value in assessing thromboembolic risk, particularly in patients with borderline CHA_2_DS_2_-VA scores.^2,167^ These echocardiographic markers have been independently associated with embolic events, reinforcing the role of atrial mechanical features in predicting such outcomes.^171^ Furthermore, when combined with CCT, TOE facilitates the evaluation of candidacy for LAA occlusion procedures—an alternative strategy for patients at elevated bleeding risk who are unsuitable for conventional anticoagulation therapy.^172^

### Cardiac CT and CMR: risk stratification and procedural planning

The role of CCT in patients with AF, initially focused on anatomical mapping prior to pulmonary vein isolation, now extends to thrombus detection, particularly in individuals unable to undergo TOE or when LAA anatomy is ambiguous.^2,167^ With appropriate delayed-phase imaging, CCT demonstrates excellent negative predictive value (>99%) for excluding LAA thrombus.^173^ While not yet endorsed by recommendations as a TOE substitute in all patients, CCT is increasingly used in high-volume centres as part of a streamlined pre-ablation work-up.^167^ In addition, CCT may be a reasonable alternative to CMR for LA fibrosis imaging, providing important prognostic information and guidance of LA catheter ablation, with good correlation with low-voltage areas.^174^

CMR adds further granularity to atrial assessment. Tissue characterization is able to confirm the echocardiographic suspicion of cardiomyopathies, particularly hypertrophic phenocopies such as amyloidosis, which is notably associated with a high rate of thromboembolic events.^175^ LGE sequences remain the gold standard for atrial fibrosis quantification—a hallmark of AtCM. Data from the DECAAF studies have shown that the extent of atrial fibrosis correlates with both arrhythmia recurrence and thromboembolic risk, independently of LA size, and may represent the next frontier in individualized AF management.^54,176,177^

Both CCT and CMR enable precise quantification of LA volume and function, which is particularly valuable for identifying atrial myopathy in patients with paroxysmal AF or those with preserved LA size but impaired mechanical function.^174^

### Beyond CHA_2_DS_2_-VA: imaging-enhanced risk stratification

While the CHA_2_DS_2_-VA score has long served as the cornerstone of anticoagulation decision-making, its categorical structure imposes inherent limitations.^2,167^ A growing body of evidence suggests that imaging markers can refine thromboembolic risk assessment, particularly in intermediate-risk patients. The concept of AtCM—defined by structural, functional, or electrophysiological atrial abnormalities, with hypercoagulability as a key component of its pathophysiological milieu—is gaining recognition as a contributor to stroke risk independent of AF burden.^49^ Imaging-derived parameters that have shown promise in this context include: LA reservoir and pump function (via STE), LAA flow velocities (via TOE) and morphology (via TOE or CCT), and the extent of atrial fibrosis (via CMR). Existing evidence indicates that LA reservoir strain <20%, LAA emptying velocity <20 cm/s, and non-chicken LAA morphologies are associated with increased embolic potential.^178^

Although these variables are not yet incorporated into formal scoring systems, they may pave the way for individualized anticoagulation strategies, particularly in patients with a CHA_2_DS_2_-VA score of 1.

### Key points

Atrial fibrillation

- TTE serves as the initial imaging modality.
- TOE is central for assessing LAA thrombotic status, with high sensitivity for thrombus, SEC, and LAA dysfunction—all linked to embolic events.
- CCT supports anatomical mapping before electrophysiological procedures and can screen for atrial thrombi when TOE is unfeasible or LAA anatomy is unclear.
- CMR quantifies atrial fibrosis, which correlates independently with AF recurrence and thromboembolic risk, aiding individualized management.
- Imaging markers refine thromboembolic risk assessment, particularly in intermediate-risk patients.
- AtCM contributes to stroke risk independent of AF burden; LA reservoir strain <20%, LAA emptying velocity <20 cm/s, and non-chicken-wing LAA morphologies are associated with increased embolic potential.

## Imaging in pre-, peri-, and post-procedural guidance of AF catheter ablation

Multimodality imaging represents a key tool for procedural success in AF substrate ablation, offering complementary insights into cardiac anatomy and function.

### Pre-procedural imaging

The ESC ‘Substrate Severity’ framework^2^ highlights LA enlargement, dysfunction, and fibrosis as main determinants of AF progression. Maximal LAVi by 3D echocardiography outperforms 2D methods in predicting AF recurrence after cryoablation, with a cut off of 30 mL/m^2^ providing 90% sensitivity and 66% specificity.^179^ A rounder LA shape and a shorter, laterally rotated appendage have also been associated with higher AF recurrence rates.^180,181^

LA strain assessed by TDI predicts reverse LA remodelling after AF ablation, with higher baseline values associated with >15% LA volume reduction and improved strain during follow-up.^182^ Reduced STE-derived reservoir strain (<23%) relates to higher recurrence risk, particularly in paroxysmal AF,^183^ a pattern also observed with CMR-derived reservoir strain.^184^

Fibrotic regions reduce myocardial contractility and increase stiffness, impairing local atrial mechanics. Atrial fibrosis on LGE-CMR correlates with echocardiographic LA strain measures,^185^ and preoperative fibrosis burden (Utah stages) independently predicts outcomes in both persistent and paroxysmal AF, with up to 69.4% relapse in stage IV patients.^186,187^ Likewise, ≥30% LA wall enhancement is associated with poor ablation response.^188^ While the DECAAF study showed that CMR-derived fibrosis independently predicts post-ablation AF recurrence,^54^ the subsequent DECAAF II trial demonstrated that targeting fibrosis during ablation did not reduce AF events compared with pulmonary vein isolation (PVI) alone, suggesting fibrosis is a useful risk marker but not an optimal ablation target.^59^

Epicardial adipose tissue volume and thickness on CCT or CMR are strong predictors of AF recurrence after ablation,^189^ with higher adipose content correlating with fibrosis and electrical dispersion. Increased LA adipose tissue attenuation shows a comparable association.^190,191^

### Intra-procedural imaging

Low voltages by electro-anatomical mapping serve as surrogate indicators of atrial fibrosis, but the precision of these measurements is often insufficient.^192^ Integrating pre-procedural imaging with electro-anatomical mapping reduces fluoroscopy time, shortens procedures, and may improve outcomes.^193,194^

LGE-CMR has been explored as a tool for guiding ablation in relation to the atrial electrical substrate. Voltage is significantly reduced in LGE-positive regions, although LGE intensity does not clearly correlate with electrogram duration, conduction abnormalities, or LA remodelling indices.^195^ These areas can be marked on electro-anatomical maps and targeted with standard ablation, either alone, in combination with PVI, or as a part of box-isolation strategies for fibrotic zones.^196^ In the ALICIA trial, CMR-guided ablation combined with PVI did not demonstrate superiority over PVI alone in preventing AF recurrences at 12 months.^60^ Conversely, another observational study in persistent AF reported that targeting fragmented LGE regions improved AF termination and reduced arrhythmia return at 1 year compared with PVI.^197^

CT quantification of epicardial fat has also been used to guide ablation strategies. PVI followed by epicardial adipose tissue ablation in patients with persistent AF resulted in a marked reduction of high-frequency sources and a 78% freedom from AF on antiarrhythmic drugs at 16-month follow-up.^198^ Benefits of these adjunctive imaging approaches on patient-reported and long-term outcomes require confirmation in clinical trials.

### Post-procedural imaging

CMR and CCT are valuable tools for evaluating post-ablation changes, including lesion formation, alterations in LA geometry, pulmonary vein stenosis, and—specifically for CMR—scar burden. While a correlation exists between LGE burden and arrhythmia recurrence, the inaccuracy and suboptimal reproducibility of quantifying atrial fibrosis by this technique limits its broader clinical application as a prognostic marker.^199^ CMR studies consistently show LA volume reduction post-ablation, independent of arrhythmia relapse, suggesting these changes are driven by the ablation itself rather than reverse remodelling.^200^ Atrial strain parameters, particularly reservoir strain, often improve within months after successful PVI, with broadly comparable effects across radiofrequency and cryoballoon techniques.^201^ Emerging data suggest that pulsed field ablation may better preserve LA function, although long-term evidence is still limited.^202^ Ablation-related scarring may reduce atrial compliance and contractile reserve contributing to the decline in LA ejection fraction.^203^

Post-ablation assessment of LGE burden can support repeat ablation by identifying veno-atrial gaps, which may lead to shorter procedures and improved outcomes. However, limitations in LGE sensitivity and variability in atrial anatomy pose challenges to consistent lesion evaluation.^204–206^

### Key points

Atrial fibrillation ablation

- Imaging markers of LA size, function, and fibrosis predict AF ablation success.
- 3D echo LAVI >30 mL/m^2^, LA reservoir strain <23%, LA LGE >30%, and larger CMR/CT epicardial fat are associated with higher AF relapse rates.
- CMR-derived fibrosis is a useful marker of AF recurrence risk but not an optimal ablation target.
- Voltage is significantly reduced in LGE-positive regions, suggesting AF substrate, but studies comparing CMR-guided ablation plus PVI versus PVI alone show equivocal results.
- CMR and CCT are valuable for assessing post-ablation changes, including lesion formation, LA geometric alterations, and pulmonary vein stenosis.
- Post-ablation LGE burden on CMR correlates with AF recurrence; however, its prognostic utility is limited by inaccuracy and suboptimal reproducibility.
- LA volume decreases after ablation irrespective of arrhythmia relapse; evidence on ablation technique-dependent improvements in atrial strain requires further validation.

## Imaging algorithms for clinical practice

### Preclinical and clinical heart failure

**Figure 6:**
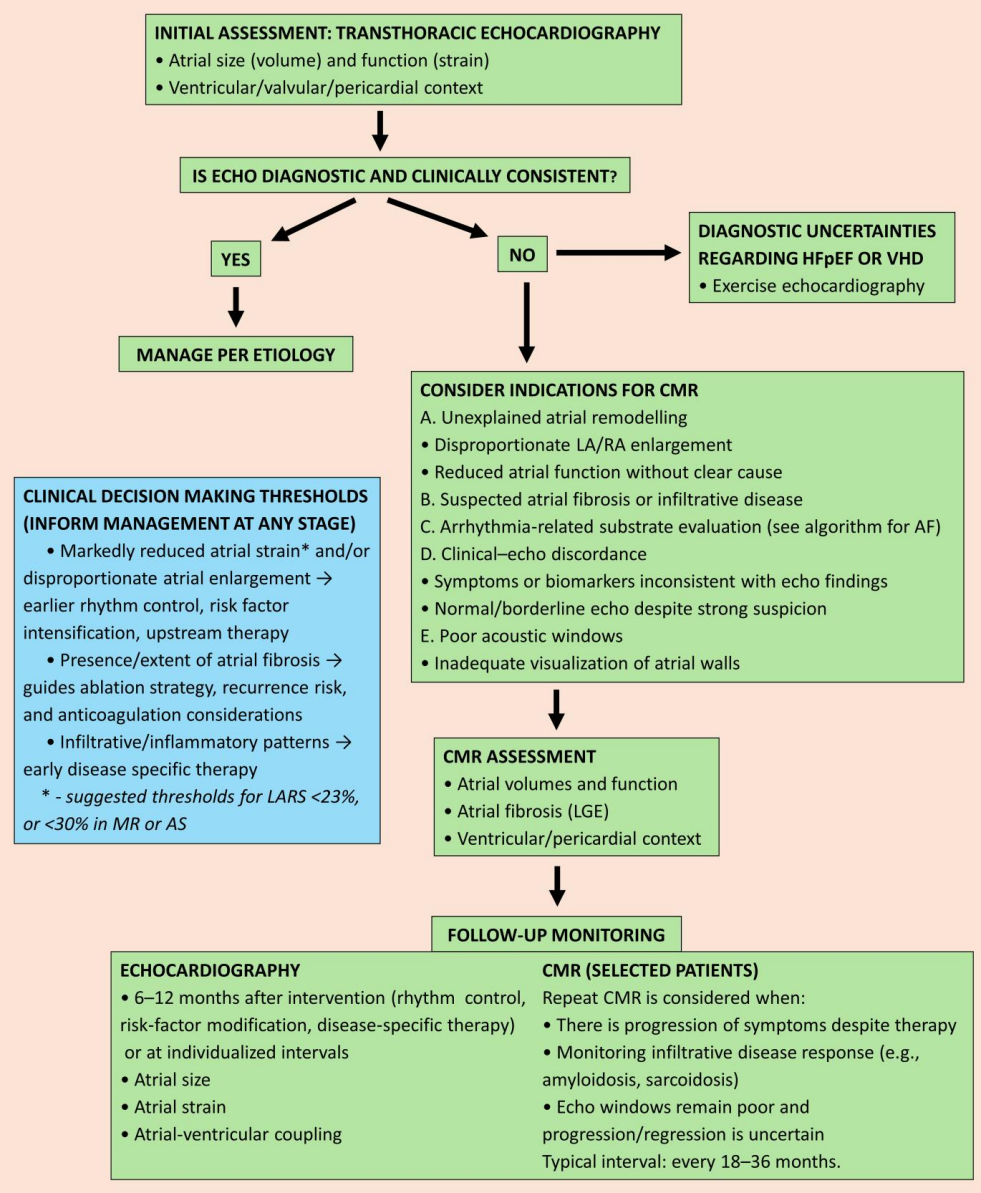
Imaging algorithm for monitoring AtCM in preclinical and clinical heart failure. AF, atrial fibrillation; AS, aortic stenosis; CMR, cardiac magnetic resonance; LA, left atrial; LARS, left atrial reservoir strain; LGE, late gadolinium enhancement; MR, mitral regurgitation; RA, right atrial; VHD, valvular heart disease

A stepwise imaging approach to AtCM assessment in preclinical and clinical HF should begin with TTE, with CMR as sequential alternative when findings are inconclusive, when atrial enlargement or dysfunction is unexplained, when infiltrative or fibrotic atrial disease is suspected, when clinical–echo discordance persists, when arrhythmia-related substrate assessment is required, or when acoustic windows are inadequate. Stress (exercise) echocardiography may be helpful in inconclusive cases of HFpEF or VHD. Importantly, clinical decision-making thresholds—such as markedly reduced atrial strain, the presence or burden of atrial fibrosis on CMR, evidence of infiltrative or inflammatory disease, or disproportionate atrial enlargement, with acknowledgment that validation of these thresholds remains suboptimal—should function as a parallel interpretive layer that can prompt earlier therapeutic interventions at any stage of the diagnostic pathway. Serial imaging, integrating echocardiography and CMR in selected patients, may help monitor disease progression and treatment responses, thereby supporting a precision-medicine framework aimed at improving patient outcomes (*Figure 6*).

### Atrial fibrillation and thromboembolic risk

**Figure 7:**
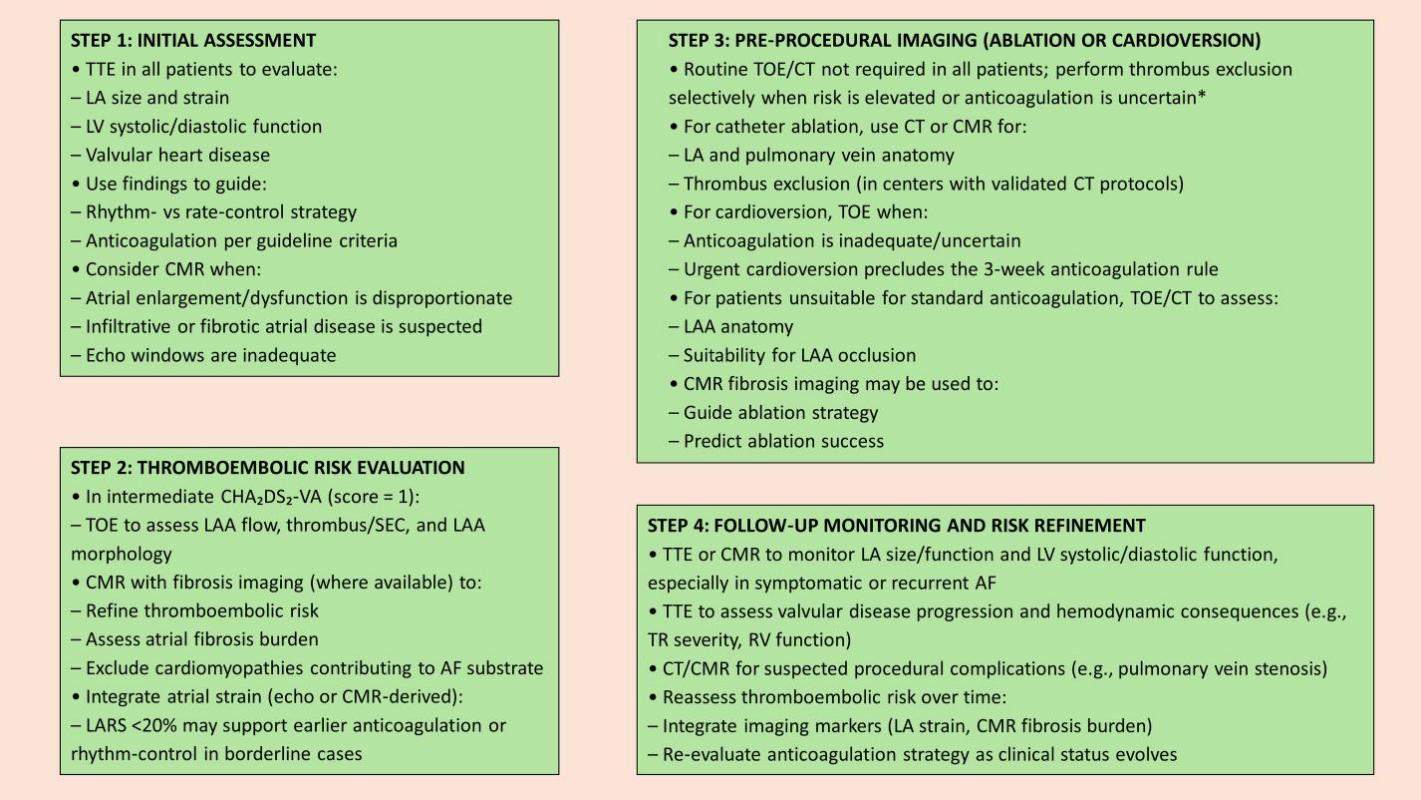
Imaging algorithm for monitoring AtCM in atrial fibrillation. AF, atrial fibrillation; CMR, cardiac magnetic resonance; CCT, cardiac computed tomography; LA, left atrial; LAA, left atrial appendage; LARS, left atrial reservoir strain; LV, left ventricular; RV, right ventricular; SEC, spontaneous echo contrast; TOE, transoesophageal echocardiography; TR, tricuspid regurgitation; TTE, transthoracic echocardiography. *—Indications for pre-procedural LA thrombus exclusion: Suboptimal anticoagulation (missed DOAC doses, INR instability). Anticoagulation duration <3 weeks before the planned procedure. High thromboembolic risk (e.g. CHA_2_DS_2_-VA ≥ 2, prior stroke/TIA, significant LA enlargement, low LAA velocities). Persistent or long-standing persistent AF. Clinical suspicion of thrombus or prior SEC. Urgent cardioversion without adequate anticoagulation

Imaging-based monitoring of AtCM in AF relies first on TTE in all patients to assess LA size and strain, LV systolic and diastolic function, and the presence or severity of VHD, thereby informing initial decisions regarding rhythm versus rate control and anticoagulation in accordance with current guideline criteria. In patients with intermediate CHA_2_DS_2_-VA score of 1, TOE may refine thromboembolic risk assessment by evaluating LAA flow velocities, thrombus, SEC, and LAA morphology. Where available, CMR with fibrosis imaging can further enhance risk stratification and exclude underlying cardiomyopathies. Markedly reduced LARS (<20%) may support earlier anticoagulation or rhythm-control strategies in borderline cases. Pre-procedural imaging should be tailored: CCT or CMR for LA and pulmonary vein anatomy; TOE before cardioversion or TOE or CCT before ablation—when anticoagulation is inadequate or timing is uncertain to exclude thrombus; and TOE or CCT to evaluate LAA anatomy in patients considered for appendage occlusion procedures. Follow-up monitoring based on TTE or CMR aims to assess atrial and ventricular function, evaluate progression of valvular disease, and detect procedural complications such as pulmonary vein stenosis, with longitudinal reassessment of thromboembolic risk incorporating imaging markers including atrial strain and fibrosis burden (*Figure 7*).

## Knowledge gaps in the application of atrial imaging to AtCM monitoring

1. Absence of a universally accepted consensus on imaging-based diagnostic criteria for AtCM and atrial failure. The field lacks an integrated, evidence-based framework for staging disease across different imaging modalities.
2. Limited longitudinal data on the prognostic relevance of atrial imaging markers used in an integrated approach.
3. Unclear algorithms for incorporating imaging findings into clinical decision-making, particularly with respect to lifestyle interventions, pharmacotherapy, and procedural planning for catheter ablation.
4. Insufficient data to support inclusion of atrial imaging in thromboembolic risk assessment strategies, despite promising insights from atrial strain and fibrosis studies.^207,208^
5. Limited standardization of imaging acquisition protocols, software platforms, and threshold values across vendors, impeding the development and validation of imaging-based monitoring algorithms.

## Conclusions and perspectives

AtCM is conceptually established as a separate entity, but its clinical significance requires further validation. Imaging remains central to clinical decision-making in this condition, and emerging evidence supports incorporating atrial abnormalities consistent with AtCM into management strategies, followed by surveillance of changes in the atrial pathological substrate. Despite growing interest, longitudinal data on the prognostic utility of AtCM remain limited. Accordingly, imaging algorithms for monitoring this condition must extend beyond the intuitive reliance on echocardiography. Although this approach continues to serve as the first-line modality due to its accessibility and cost-effectiveness, integration with advanced techniques such as CMR and CCT provides incremental value in selected patients.

Atrial imaging workflows in AF—particularly for rhythm-control interventions—are becoming increasingly standardized, but AtCM-focused pathways in other clinical settings remain underdeveloped. Embedding structured, multimodal imaging into routine practice offers the potential to enhance early detection, individualize therapeutic strategies, and improve long-term outcomes in patients with AtCM and atrial failure. Such integration should be supported by uniform protocols and validated imaging criteria applicable across a spectrum of disease severities and aetiologies. However, important gaps persist, including the need for standardized imaging thresholds to distinguish adverse from reverse remodelling, optimal monitoring intervals, cost-effectiveness analyses, and phenotype-specific surveillance strategies tailored to AtCM subtypes.

This consensus paper synthesizes current evidence and proposes an imaging-based monitoring framework for AtCM, setting the stage for future refinement and clinical implementation. By promoting a more unified and evidence-driven approach to atrial imaging, the proposed framework may help advance the field toward more precise and personalized management of atrial disease.

## Declarations

### Data Availability

No data were generated or analysed for this manuscript.
